# WeatherChimes: An Open IoT Weather Station and Data Sonification System

**DOI:** 10.1016/j.ohx.2023.e00402

**Published:** 2023-02-16

**Authors:** Winnie Woo, William Richards, John Selker, Chet Udell

**Affiliations:** OPEnS Lab, Oregon State University, Corvallis, OR, United States

**Keywords:** Environmental Observation, Data Logging, Arduino, Data Sonification, MQTT, MongoDB, Near real-time Data, Max8

## Abstract

Many people in the United States are disconnected from their environment: urban residents spend 90% of their time indoors inside confined climate-controlled spaces. In addition to being physically separated from the natural environment, much of human understanding of the world’s environment is inferred from data collected by satellites orbiting 22,000 miles away. In contrast, in-situ environmental sensor systems are physically accessible, location specific, and essential for correcting and validating weather measurements. However, present options for in-situ systems are mostly limited to expensive, proprietary commercial data loggers with inflexible data access protocols. WeatherChimes is an open-source Arduino-programmable, low-cost hardware and software suite that enables near real-time access to in-situ environmental sensor data (including light, temperature, relative humidity, and soil moisture) anywhere with a WiFi internet connection. Scientists, educators, and artists alike can use this tool to obtain and interact with environmental data in new and innovative ways, as well as collaborate remotely. Transforming data collection processes of environmental sensors into Internet of Things (IoT) compatible formats opens new doors into accessing, understanding, and interacting with natural phenomena. WeatherChimes not only enables users to observe data online, but can also transform data into auditory signals and soundscapes through sonification processes or creative animations using newly-created computer applications. Lab and field tests have confirmed the sensor and online data logging performance of the system. We describe the application of WeatherChimes in an undergraduate Honors College classroom and STEM (Science, Technology, Engineering, and Math) education workshop series in Sitka Alaska, which was used to not only teach about environmental sensors, but to explore how different aspects of our environment are interrelated (e.g. temperature and humidity) through sonification.

**Specifications table:** 

Hardware Name	WeatherChimes
Subject Area	Environmental, Planetary and Agricultural sciences
Hardware Type	Field Measurements and Sensors
Closest Commercial Analog	IoT Weather Station
Open Source License	CERN Open Hardware License GNU General Public License v3.0
Cost of Hardware	$530
Source File Repository	https://doi.org/10.5281/zenodo.7328528

## Hardware in context

The need for, and lack of, distributed in-situ environmental sensor networks is well documented [1], [2], [3]. Environmental sensors are essential for providing near real-time data to monitor weather and other environmental phenomena that cannot be accomplished remotely via satellite [4]. The list of open source environmental sensor systems continues to expand with increasing availability of components and resources. ALog BottleLogger by Northern Widget LLC is an open field sensor data acquisition system, but lacks telemetry [5]. MayFly by EnviroDIY is an open Arduino based system that offers telemetry options including Xbee, LoRa, and 4G, but the sensors focus primarily on water applications [6]. The ThingsBoard is an open IoT rapid development platform for the management and scaling of sensor projects, but relies on RaspberryPi hardware, a versatile and tiny computer with an operating system that is not optimized for low-power, minimal-peripheral field sensor applications [7]. WeatherChimes is low-cost ($530, including all components and high-quality sensors and $254 without the sensors), low-power, and has the capacity to accommodate a variety of other sensors across broad applications. Compared to commercial IoT weather stations, WeatherChimes is up to 70 % cheaper than the competitor products [8], [9], [10]. It is also lightweight (weighs 1.71 lbs compared to commercial systems weighing 16 lbs) and smaller than other devices, making it much more portable and easier to set up.

Each *Chime* can measure soil moisture, soil electrical conductivity, and soil temperature (Meter GS3); air temperature and humidity (SHT31/SHT30); and solar luminosity (TSL2591), and log data at user defined intervals to the cloud database: MongoDB. Beyond the sensors used in this paper, the *Chime* is capable of using a variety of analog, digital, I2C, SDI-12, and other serial sensors via footprints on the printed circuit board (PCB) detailed in the sections below. While many other sensors like rainfall, air quality, and wind direction could have been chosen, we selected the current combination of sensors to cover a proof of concept with broad applications using only a few sensors to start. Temperature, relative humidity, solar irradiance, and soil moisture can be combined using a variety of well-known methods to derive other environmental metrics. For example, the Penman–Monteith equation can be adapted to translate the above environmental metrics to approximate net evapotranspiration, which has applications from watershed water balance to crop water transfer from soil into the atmosphere via plants (transpiration) and evaporation [11], [12], [13], [14]. Because the relationships between these environmental parameters are well-studied, if one knows the soil moisture and temperature, air temperature and humidity, and solar irradiance, one could approximate aspects like wind speed. Other sensors may be substituted, added, or removed to meet particular applications, and we hope to conduct user experience surveys moving forward to inform these future decisions. While environmental parameters like temperature may not change rapidly and in different directions throughout the day, solar irradiance and soil moisture due to weather events might. For the purposes of our proof of concept, we determined ten minutes was a good trade-off between real-time data and power consumption to catch fluctuations in light and soil moisture (as an indicator of rainfall events) throughout the daytime. The *Chime* can operate for up to 27 days on a battery capacity of 10,050 mAh with a logging period of every ten minutes. The logging period is arbitrary and can be adjusted to accommodate any power requirements. The total operation duration of the system can be lengthened significantly with the addition of a solar panel and better power management, which we hope to achieve in the next iteration. In this proof of concept, we present the IoT hardware and software system as well as examples of data sonification and visualization tools, like the software platform Max8 by Cycling74.

WeatherChimes logs real-time sensor data to an internal SD memory card and via network to the MongoDB online database. Typical IoT systems conclude their operation chain at the database layer for plotting and downloading data reports. WeatherChimes goes a step further by incorporating novel data sonification and visualization applications that pull data from MongoDB using NodeJS into a platform called Max8. Although the main focus of this paper is on the WeatherChimes hardware and its construction, it is important to note the significant context of data sonification and visualization applications on which WeatherChimes was conceived.

The lack of exposure, and awareness, of the unseen and unheard environmental processes that occur both outside our own window and over remote distances during periods beyond the average human's attention span could be a limiting factor regarding the connection between humans and nature in our modern society. Many people who live in urban areas spend the majority of their time in the confines of a regulated, indoor environment [15], [16]. Wind chimes are an excellent example of something that translates an unseen environmental phenomenon into an observable, informative, and aesthetic signal. Our awareness of the natural processes outside our window can be enhanced just by hearing the sound of the chimes without the need for visual attention. WeatherChimes stems from this idea, and follows ample examples of environmental data sonification and alternative visualization projects and strategies.

Utilizing environmental data as a means for artistic interaction is still somewhat novel and underexplored, but has a growing tradition. The sonification of data allows researchers to detect new patterns and structures, particularly those that emerge over periods of time, in ways that may not be obvious to our eyes when interpreting a graph at a glance [17]. Traditional visual data displays may be effective for monitoring and identifying trends and features, but require our undivided attention. Auditory “displays” like heart rate monitors enable us to perceive important information in the background or from another room. As an alternative to traditional data representations, data can be visualized and sonified with artistic or communicative purposes in mind to allow a broader audience to explore data and experience phenomena through creative expression.

A Hubbard Brook Ecosystem study in 2004 titled WaterViz [18] was a data visualization and sonification project involving hydrologic data from Hubbard Brook Experimental Forest, an NSF LTER watershed in New Hampshire, and generated a computer model with artistic and musical simulations of the water cycle in real-time. The purpose of the project was to provide a tool for students and educators to visualize how the water cycle works in a forest and how it is affected by human activities. In a similar context, WeatherChimes allows for an educational tone regarding the artistic design of more scientific data. By providing different mediums to interpret in-depth studies, scientists can team-up with artists to educate and inform the public about the hidden layers of nature all around them.

Weather Works [19], by environmental artist and writer Andrea Polli, was a storm sonification project, and is a great example of sonification in action. The 2004 study took meteorological data, such as atmospheric pressure, temperature, and humidity, from storms on the East Coast and created a model of the conditions at various elevations. The framework of this project in regards to its sonification of meteorological data is similar to WeatherChimes, but the technology that was used to acquire the sonified data set was not something that could be feasibly replicated by the average user. WeatherChimes strives to bridge the gap between high-level scientific data collection and user-friendly, creative platforms. Providing intentionally designed tools for seamless functionality with the WeatherChimes project, via the use of MongoDB and Max8 applications, allows individuals to jump into the creative process for which this device was designed.

The ability to remotely obtain both local and real-time environmental sensor data is a unique feature that many low-cost, proprietary devices do not offer. WeatherChimes goes beyond data collection and provides users with the ability to transform data from the soil, air and sun into alternative sensory signals. Scientists and artists alike will continue to gain new insights into our ever-changing environment aided by cloud-based storage. Additionally, the use of third-party applications transforms data into musical, visual, and other artistic representations of the environment for academic, educational, and entertainment purposes.

## Hardware description

### Hardware components

**Fig. 1:**
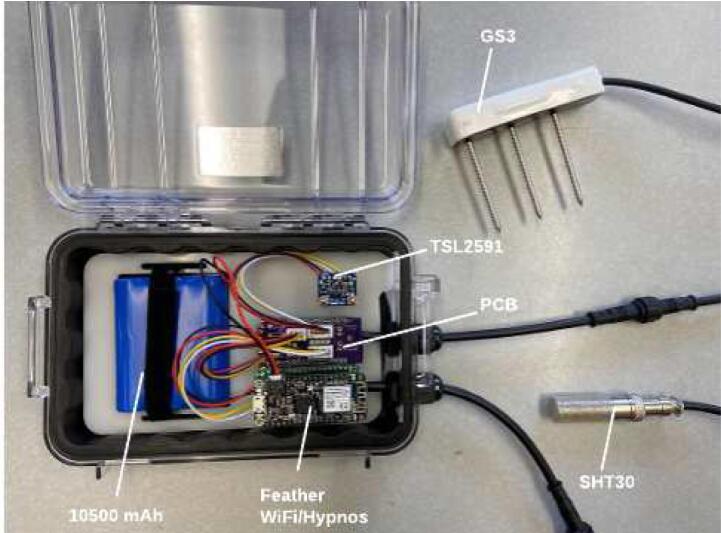
Completed WeatherChimes device.

The WeatherChimes device (Fig. 1) is housed in a modified Pelican 1040 case with 2 of the sensors deployed externally. Inside the case, a custom base plate made from 1/8″ acrylic sheet holds an Adafruit Featherwing doubler (to easily connect components) with stacked Printed Circuit Boards (PCBs), light sensor (TSL2591), and battery with fasteners. A custom PCB, Chime V1, contains headers and connections for the sensors including I2C (TSL2591, SHT30) and SDI12 (GS3) connections. Other I2C and SDI12 sensors may also be connected as long as there is relevant code to handle requesting data on the Feather M0. There are footprints for other components included on the PCB for future utility including a push button, audio jack, and 3-pin JST receptacles for analog signals. These are not used in this version of the project.

### Electronics

The Feather M0 WiFi PCB [20] microcontroller used to run the system program in the sensor package and transmit data via WiFi. While the Hypnos v3.2 PCB designed and openly-published by the OPEnS Lab [21] is used to turn peripherals on and off to preserve power, wake up at intervals using the embedded DS3231 RTC, and store data to an onboard SD card.

**Fig. 2:**
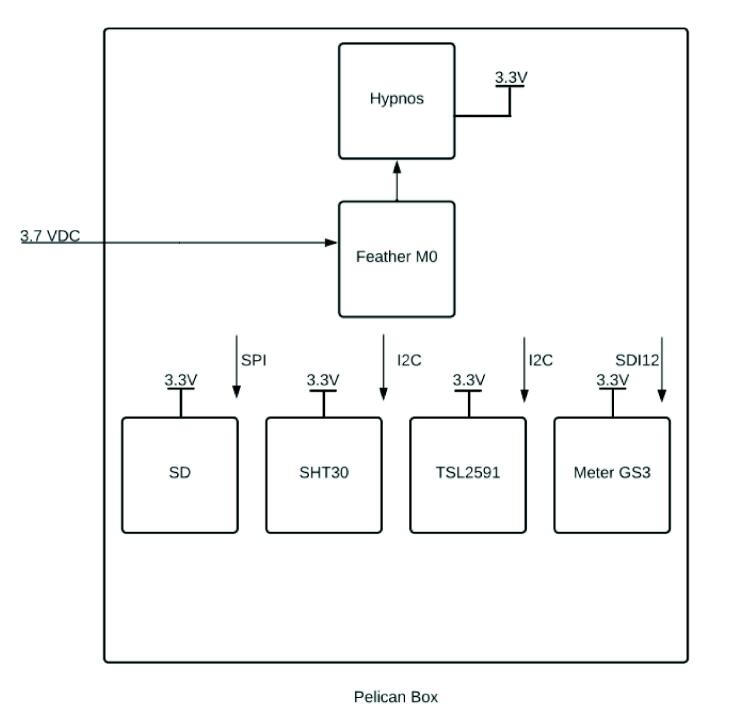
Block diagram of WeatherChimes electronics.

The sensors used include a luminosity sensor to derive solar radiation (TSL2591 [22]), a temperature and humidity sensor (SHT30 [23]), and a subterranean soil moisture, temperature, and electrical conductivity sensor (Meter GS3 [24]). The Feather M0 is connected to the 3.7V LiPolymer battery, which is then connected to the Hypnos board for 3.3 V power switching. The sensors are then linked to the Hypnos for 3.3V power (Fig. 2).

A Feather M0 WiFi board, an Arduino programmable microcontroller with internet connectivity, was used because of the availability of open WiFi networks for the proof of concept deployment on the Oregon State University (OSU) campus. This was the most cost-effective option compared to other available methods to establish a network connection including 4G. It is understood that access to a WiFi connection is exceptional for outdoor field applications, and we are investigating the implementation of a 4G cellular network connection in lieu of a WiFi in an upcoming version.

In addition, WeatherChimes uses a data-base logging service that does not require a subscription fee and is accessible from anywhere with an internet connection. The majority of weather station devices do not offer the option of sonification and visualization applications (beyond plotting on a graph). WeatherChimes provides users with an efficient and engaging way to explore large datasets from many sensory modalities, enabling broader audiences to experience the data in different ways.

The most relevant features of the electronics system are the following:

● Measures soil dielectric (volumetric water content), electrical conductivity, temperature
● Measures air temperature and humidity
● Measures infrared, full spectrum, and visible light
● Saves data to SD
● Onboard RTC and power switching relays for power savings
● WiFi access to upload data to MongoDB server

### Data handling

**Fig. 3:**
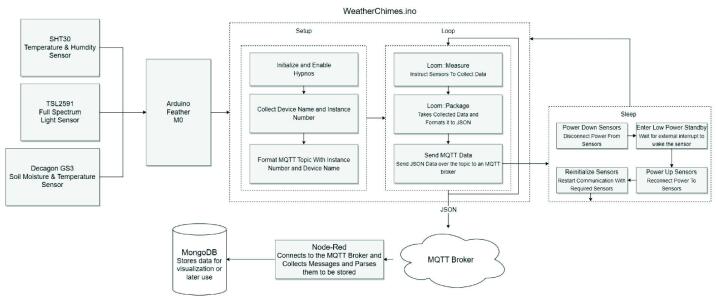
Data handling flowchart for WeatherChimes.

Each sample cycle is triggered by RTC alarm to wake from a low-power sleep mode, the Feather M0 requests data from each of the sensors with the Loom Measure code and formats the data according to each logging platform: comma separated for local storage on microSD and JSON for telemetry. After all sensor information has been collected and formatted, the Feather will initiate a message over WiFi to a remote MQTT (Message Queueing Telemetry Transport) broker which is being run on an OSU server (Fig. 3).

MQTT brokers work by utilizing a publish/subscribe paradigm, this paradigm works on the basis that there are “topics” that are public to every-one viewing the broker. Users can subscribe to topics which allows them to receive a callback when new data is published to the topic. For WeatherChimes, all data messages are sent over a topic, the topic is formatted with the “Site Name”/”Device Name” + “Device Number” to distinguish between the devices and their locations and determine the destination, i.e. collection, in the MongoDB database. Assigning a two part topic to each message allows multiple devices, even with the same name, to publish to different collections of data.

**Fig. 4:**
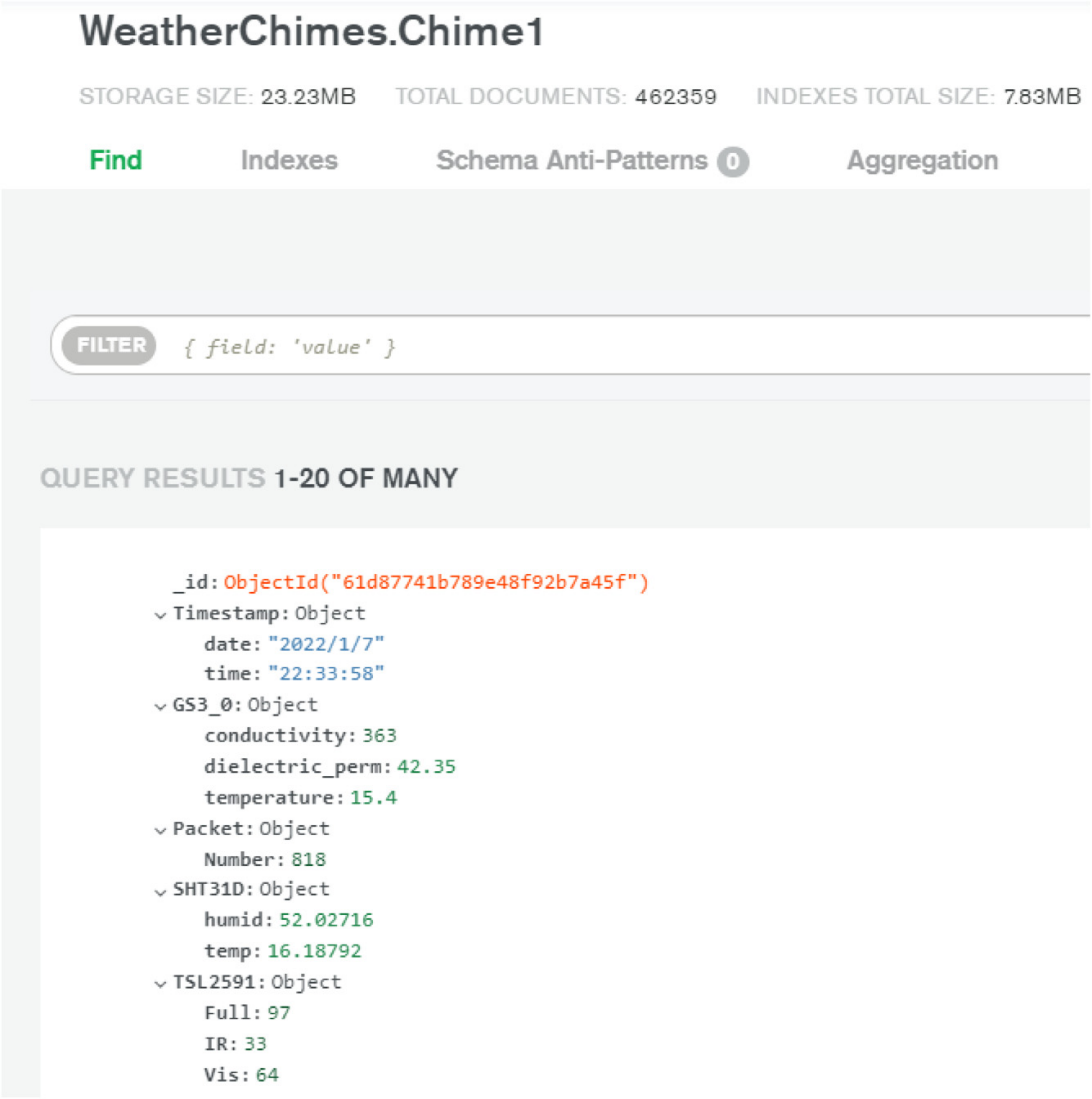
Example of single instance of data, document, in MongoDB from Chime 1.

Once the data has been received by the MQTT broker, a listener hosted by the program Node-RED manages the passthrough of data from the MQTT broker to the MongoDB cloud database. Node-RED is a programming tool for IoT devices that supports seamless communication between online services. Using this passthrough also allows us to have intermediary steps where we are able to parse the data to be formatted in an easy to use format. Upon receipt by MongoDB, the *Site Name* and *Device Name + Number*, the topic, are parsed. The data is then pushed into a new packet/document within a MongoDB collection containing all received on that topic (Fig. 4).

Once stored within a MongoDB collection, the Max 8 application can be used to subscribe to the data. We programmed a NodeJS application that handles all communication, subscription, and parsing of data between Max 8 and MongoDB database. This allows users to pull up past data as well as receive real-time updates from the WeatherChimes device, opening up numerous possibilities for manipulating and utilizing the data including downloading, sonification, and visualization.

## Design files

*Design files summary*

**Table:** 

Design file name	File type	Open source license	Location of the file
Base Plate	STL	CERN Open Hardware License	https://doi.org/10.5281/zenodo.7328528
ChimeV1	BRD	CERN Open Hardware License	https://doi.org/10.5281/zenodo.7328528
WeatherChimes	ino	GNU General Public License v3.0	https://doi.org/10.5281/zenodo.7328528
config	header	GNU General Public License v3.0	https://doi.org/10.5281/zenodo.7328528
arduino_secrets	header	GNU General Public License v3.0	https://doi.org/10.5281/zenodo.7328528
MQTT	header	GNU General Public License v3.0	https://doi.org/10.5281/zenodo.7328528
MQTT2Mongo	js	GNU General Public License v3.0	https://doi.org/10.5281/zenodo.7328528
Mongo2Max**	js	GNU General Public License v3.0	https://doi.org/10.5281/zenodo.7328528
mongodb**	maxpat	GNU General Public License v3.0	https://doi.org/10.5281/zenodo.7328528
.config.nodes*	js	GNU General Public License v3.0	https://doi.org/10.5281/zenodo.7328528
.config.nodes.json.backup*	js	GNU General Public License v3.0	https://doi.org/10.5281/zenodo.7328528
.config.runtime*	js	GNU General Public License v3.0	https://doi.org/10.5281/zenodo.7328528
.config.users*	js	GNU General Public License v3.0	https://doi.org/10.5281/zenodo.7328528
.config.users.json.backup*	js	GNU General Public License v3.0	https://doi.org/10.5281/zenodo.7328528
.flows.json.backup*	js	GNU General Public License v3.0	https://doi.org/10.5281/zenodo.7328528
.flows_cred.json.backup*	js	GNU General Public License v3.0	https://doi.org/10.5281/zenodo.7328528
flows.json*	js	GNU General Public License v3.0	https://doi.org/10.5281/zenodo.7328528
flows_cred.json*	js	GNU General Public License v3.0	https://doi.org/10.5281/zenodo.7328528
settings.js*	js	GNU General Public License v3.0	https://doi.org/10.5281/zenodo.7328528

**Base Plate**: STL file for 3D printing the insert which holds the electronics inside the Pelican case.

**ChimeV1**: BRD file for creating the I2C multiplexer printed circuit board (PCB).

**WeatherChimes**: Arduino file used as firmware for a microcontroller.

**Config**: Header file used as firmware for a microcontroller.

**Arduino_secrets**: Header file containing WiFi and MQTT settings.

**MQTT**: header file containing MQTT settings.

**Mongo2Max: JavaScript helper function for the patch should be placed within the javascript subfolder inside the Loom folder for Max.

**mongodb: Patcher for within Max8 itself.

*NodeRed: Flows and charts for processing MQTT packets.

## Bill of materials

**Table:** 

**Designator**	**Component**	**Number of Units**	**Cost per Unit**	**Cost for Project**	**Total/Real Cost**	**Source of Material**	**Material Type**
PG7 Cable Gland	PG7 Cord Grip^2^	2	$0.90	$1.80	$8.99	Amazon	Polymer
Hex Standoff	Hex Standoff	2	$1.84	$3.68	$3.68	Digikey	Brass
2.5 mm nuts	2.5 mm nuts**^1^**	6	$0.01	$0.06	$11.99	Amazon	Steel
2.5 mm screws	2.5 mm screws**^1^**	4	$0.01	$0.04	$11.99	Amazon	Steel
Pelican Case	Pelican 1040	1	$14.95	$14.95	$14.95	Amazon	Polymer
JST XH	4-pin JST^2^	2	$0.38	$0.76	$7.59	Amazon	Non-specific
JST XH	3-pin JST^3^	1	$0.35	$0.35	$6.99	Amazon	Non-specific
Hook-and-Loop	Hook-and-Loop tie-down	1	$7.99	$7.99	$7.99	Amazon	Nylon
Silicon Coating	422C-55MLCA	1	$26.43	$26.43	$26.43	Amazon	Non-specific
Case Insert	Acrylic sheet^6^	1	$5.70	$5.70	$5.70	McMaster	Acrylic Plastic
Small diameter heat shrink	Heat shrink^4^	14	$0.02	$0.28	$7.29	Amazon	Polyolefin
Large diameter heat shrink	Heat shrink^4^	4	$0.02	$0.08	$7.29	Amazon	Polyolefin
Desiccant	Desiccant	2	$0.16	$0.32	$6.99	Amazon	Silica gel
Coin Cell	Coin Cell	1	$0.95	$0.95	$0.95	Mouser	Non-specific
Female Headers	Female Headers	2	$0.90	$1.90	$1.90	Adafruit	Non-specific
Male Headers	Male Headers	3	$0.50	$1.50	$1.50	Adafruit	Non-specific
Proto Doubler	Proto Doubler	1	$7.50	$7.50	$7.50	Adafruit	Non-specific
Cable Set	Cable Set	2	$2.50	$5.00	$5.00	Adafruit	Non-specific
Feather M0 WiFi	Feather M0 WiFi	1	$34.95	$34.95	$34.95	Adafruit	Non-specific
SD Card	SD Card	1	$7.99	$7.99	$7.99	Amazon	Non-specific
SHT30	SHT30	1	$24.95	$24.95	$24.95	Adafruit	Non-specific
TSL2591	TSL2591	1	$6.95	$6.95	$6.95	Adafruit	Non-specific
Teros 12	Teros 12	1	$246.00	$246.00	$246.00	Meter Group	Non-specific
10,050 mAh battery	10,050 mAh battery	1	$29.95	$29.95	$29.95	Adafruit	Non-specific
PCB	PCB^5^	1	$2.96	$2.96	$8.90	OSHPark	Non-specific
Hypnos	Hypnos Board	1	$27.00	$27.00	$27.00	OPEnS	Non-specific
1 - Sold in the same pack with 264 pieces holding 100 units each 2 - Sold in a pack of 20 units 3 - Sold in a pack of 20 units 4 - Sold in the same pack with 520 pieces holding 50 small and 30 large units 5 - Sold in multiples of 3 6 - Can make 4 base plates							

## Build instructions

### Mechanical assembly

#### PG7 cable glands

**Fig. 5:**
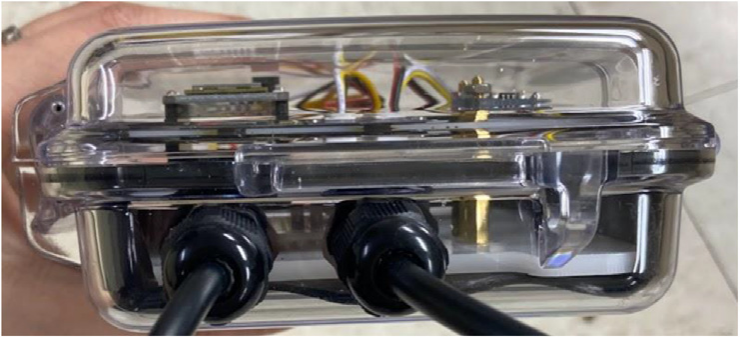
Cable Glands with cables installed into holes drilled into Pelican case.

To install the cable glands in the Pelican case, drill two holes with a 7/16″ drill bit and use a ½-13 tap to create the threads on the right side with the carabiner hole. The glands should be spaced approximately 9.5 mm from each other and 9.5 mm from the bottom curve of the case. There is no specified distance as to how far from the center the holes need to be, as long as they are not drilled too close together and too close to the edge of the case. The Pelican case should be held in a clamp, taking care not to apply too much pressure to alter the shape. Twist the ½-13 NC tapping tool in the hole for one full rotation and then backwards 1/2 a rotation; repeat this until the entire hole is threaded. Then screw in the cable gland and the nut on the cable gland from the inside (Fig. 5).

### Electrical assembly

#### Feather M0 WiFi

**Fig. 6:**
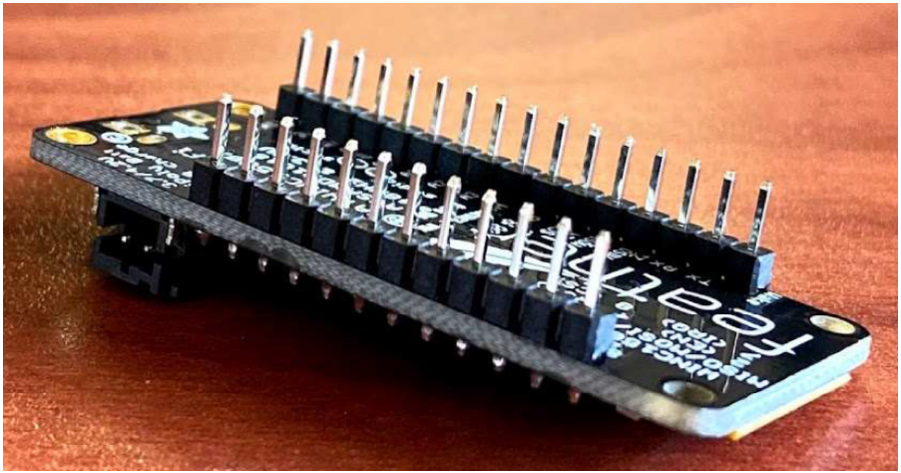
Male headers on Feather M0 WiFi.

For the Feather M0 WiFi, insert male headers from the bottom of the board with the long side facing downward, and solder into place from the top (Fig. 6). Take care not to touch the soldering tip or recently heated components, and always place the soldering iron back in its stand when not in use. Conduct soldering work in a well-ventilated area if using lead based solder. To conformally coat the Feather, place the device on a piece of cardboard. In a well-ventilated area, take a paintbrush and apply coating to the front and back of the components on the Feather, taking care not to coat the reset button or the long end of the header pins. Allow the coating to fume and harden completely for 24 hrs before using or applying a second coat.

#### Proto doubler

**Fig. 7:**
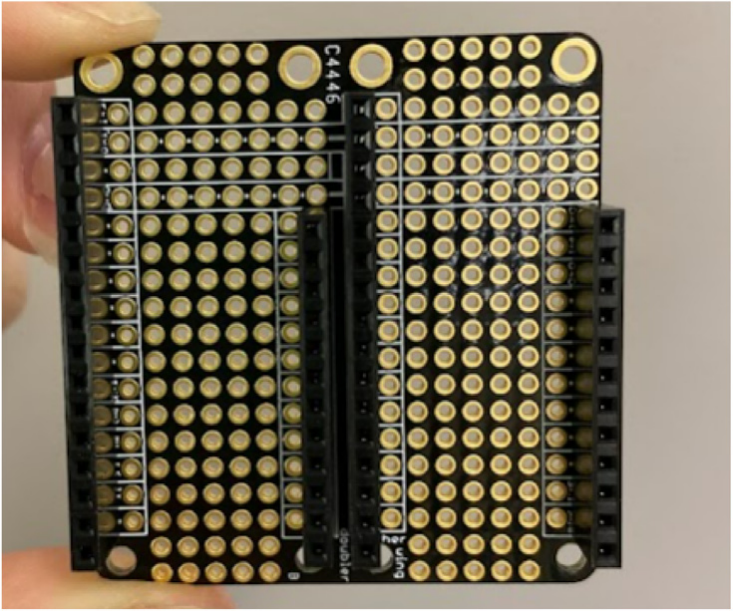
Feather doubler with two sets of female headers.

**Fig. 8:**
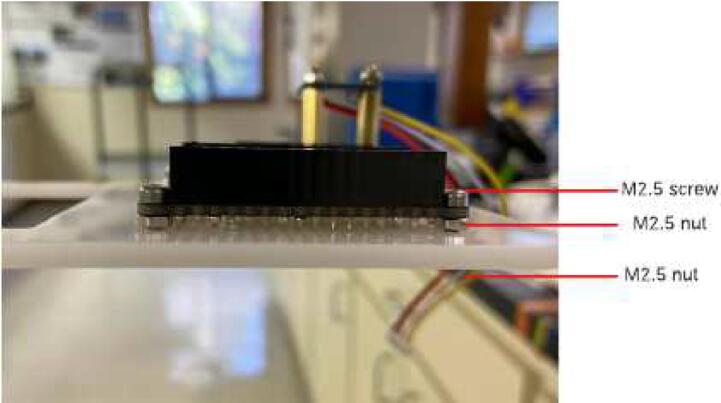
Doubler attachment to base plate.

For the doubler, solder two pairs of short female headers with a 16 pin and 12 pin header on the outer sides of the doubler and a 12 pin and 16 pin header in the middle (Fig. 7). Note that a Feather M0 board with male headers should fit on top of the headers on each side of the doubler. Attach the doubler to the case insert using M2.5 screws and M2.5 nuts. Use one set of 4 nuts between the doubler and case insert to cushion the doubler from bending and another set of 4 nuts on the bottom of the base plate (Fig. 8).

#### Hypnos

**Fig. 9:**
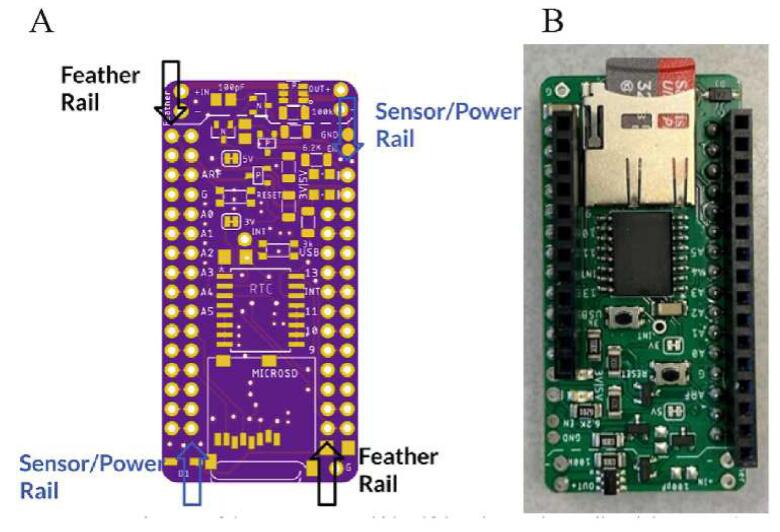
Hypnos Assembly from the top. A) Board rendering showing the Feather (black arrows) and sensor/power (blue arrows) rails. B) Completed board with male and female headers. (For interpretation of the references to color in this figure legend, the reader is referred to the web version of this article.)

A detailed guide to assembling the Hypnos Board can be found on the official Hypnos GitHub wiki [25]. The build guide covers how to place all SMD components onto the Hypnos board. After placing and soldering all SMD components, solder 12-pin female headers on the top of the board on the inner rail [Feather rail] on the right of the Hypnos front and 16-pin female headers on the outer rail on the left side (Fig. 9). Then, solder 12-pin male headers on the bottom of the board on the outer edge [sensor rail] of the right side, long side facing down and 16-pin male headers on the inner rail on the left side, long side facing down (Fig. 9). To conformal coat the Hypnos, take a paintbrush and apply coating on the front and back; only contacts and exposed metal need to be coated. Allow the coating to fume and harden for 24 h before using. Insert a coin cell battery into the slot on the bottom side of the Hypnos board, and a microSD card into the slot on the top side of the Hypnos board.

#### Chime V1 PCB

**Fig. 10:**
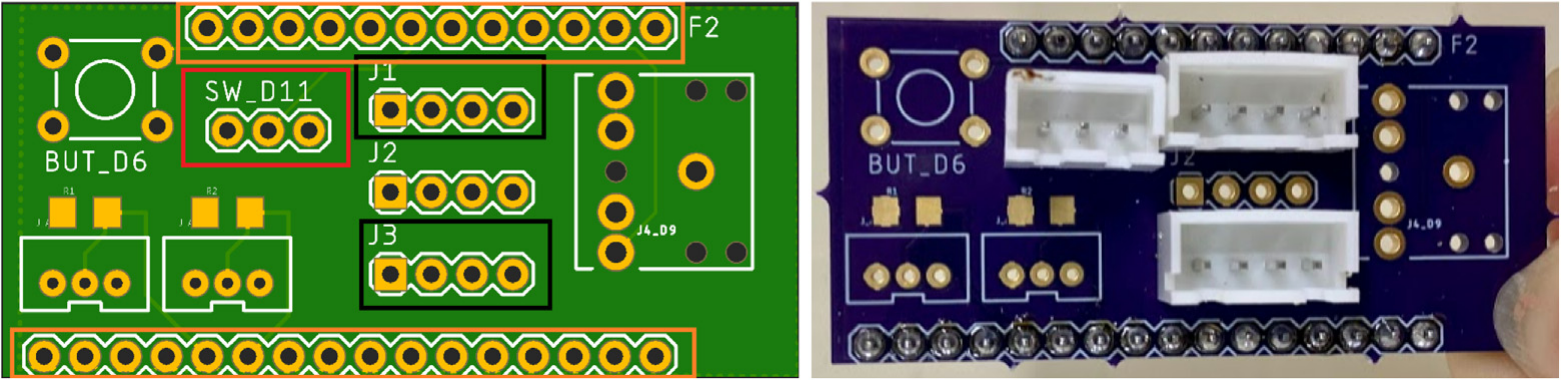
PCB from the top. Left: Board rendering showing 3-pin JST location for SDI-12 sensor (SW_D11, red box), 4-pin JST locations for i2c sensors (J1, J3, black boxes), and header (orange boxes). Right: PCB populated with JSTs and male headers. Note orientation of notches for each connector for proper wire color agreement. (For interpretation of the references to color in this figure legend, the reader is referred to the web version of this article.)

On the top side of the PCB, solder a male 3-pin JST connector (label SW_D11), and two male 4-pin JST connectors (label J1, J3) as shown in Fig. 10. Connectors should be oriented so that all notches are on the side closest to the longer line (16) of pins for the Feather header (Fig. 10, right). Then solder 12- and 16-pin male headers into the board on the Feather pins (12-pin labeled F2); insert the headers from the bottom of the board with the long side facing downward. There are footprints for other components included on the PCB for future utility including BUT_D6, J4_D9, J2 i2c, and 3-pin JST receptacles (bottom left of Fig. 10) for analog signals that we do not use in this project.

### Soil moisture sensor

In order to facilitate separating the GS3 sensor from the enclosure, a cable set with waterproof connectors is inserted between the end of the GS3 cable outside the enclosure and the end of the JST wires inside the enclosure.

#### GS3 to cable set

**Fig. 11:**
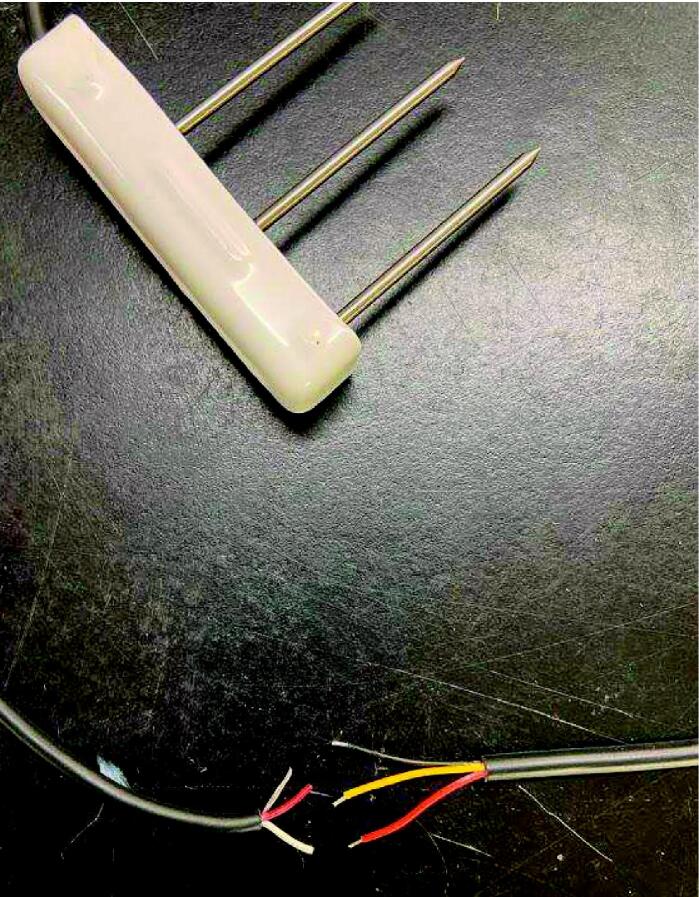
GS3 (left cable) to cable set (right cable) wiring.

Cut down the bare wire end of one of the male sides of a cable set to 61 cm long. Slide two 45 mm long large diameter heat shrink tubes over the cable to at least 10 cm away from the bare end. Strip the outer jacket of the cable at the bare end back 2–3 cm to expose the individual wire jackets. Cut off the white wire flush with the outer jacket. Strip remaining three wires back 0.5–1 cm using a wire stripper. Slide a 23 mm long piece of small diameter heat shrink over each wire to at least 1 cm away from the bare end. Using a soldering iron, connect the three wires from the GS3 cable to the cable set following the wire connection colors in Table 1 (Fig. 11). Slide each small diameter heat shrink so that it is centered over the solder joint of each individual wire pair, and shrink with a heat gun. Slide one of the large diameter heat shrink tubes over the three wires so that it is centered over the small diameter heat shrink, and shrink with a heat gun.

**Table 1:** GS3 to cable set wiring colors.

**Label**	**GS3 wire color**[9]	**Cable set wire color**
Ground	Bare	Black
Data	Red	Yellow
Power	White	Red

#### Cable set to JST

**Fig. 12:**
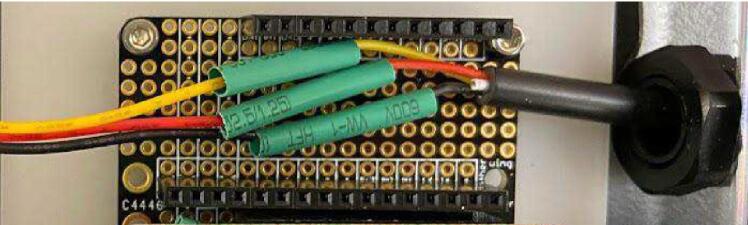
Cable set wires for GS3 sensor soldered to JST wires inside Pelican case.

Insert the bare wire end of the female side of the cable set into the Pelican case through the cable grip closest to the center of the short side of the Pelican case. Cut the white wire flush with the cable jacket. Slide a 23 mm long small diameter heat shrink tube over each wire on the JST side to at least 10 cm away from the bare end. Solder the cable set wires to the JST wires by matching colors. Slide each small diameter heat shrink so that it is centered over the solder joint of each individual wire pair, and shrink with a heat gun. Slide the cable set back out of the Pelican case through the cable gland until the cable set jacket is almost off the edge of the doubler, and tighten the cable gland (Fig. 12).

#### SHT30

For an easier separation of the SHT sensor from the enclosure, a cable set with waterproof connectors is inserted between the end of the SHT cable outside the enclosure and the end of the JST wires inside the enclosure.

#### SHT30 to cable set

Cut down the bare wire end of the SHT cable to 40 cm long. Slide two 45 mm-long large diameter heat shrink pieces over the cable to at least 10 cm away from the bare end. Strip the outer jacket of the cable at the bare end back 2–3 cm to expose the individual wire jackets. Strip the four wires back 0.5–1 cm using a wire stripper. Slide a 23 mm long small diameter heat shrink tube over each wire to at least 1 cm away from the bare end. Using a soldering iron, connect the four wires from the SHT cable to the cable set following the wire connection colors in Table 2. Slide each small diameter heat shrink so that it is centered over the solder joint of each individual wire pair, and shrink with a heat gun. Slide the large diameter heat shrink over the four wires so that it is centered over the small diameter heat shrink, and shrink with a heat gun.

**Table 2:** SHT30 to cable set wiring colors.

**Label**	**SHT30 wire color**[15]	**Cable set wire color**
Clock	Yellow	Yellow
Data	Green/Blue	White
Power (VCC)	Brown/Red	Red
Ground	Black	Black

#### Cable set to JST

**Fig. 13:**
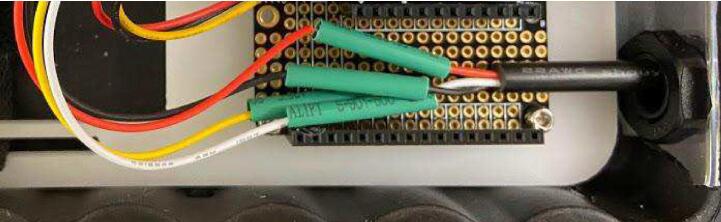
Cable set wires for SHT30 sensor soldered to JST wires inside Pelican case.

Insert the bare wire end of the female side of the cable set into the Pelican case through the cable grip farthest from the center of the short side of the Pelican case. Slide a 23 mm long small diameter heat shrink tube over each wire on the JST side to at least 10 cm away from the bare end. Solder the cable set wires to the JST wires by matching colors. Slide each small diameter heat shrink tube so that it is centered over the solder joint of each individual wire pair, and shrink with a heat gun. Slide the cable set back out of the Pelican case through the cable gland until the cable set jacket is almost off the edge of the doubler, and tighten the cable gland (Fig. 13).

#### TSL2591

**Fig. 14:**
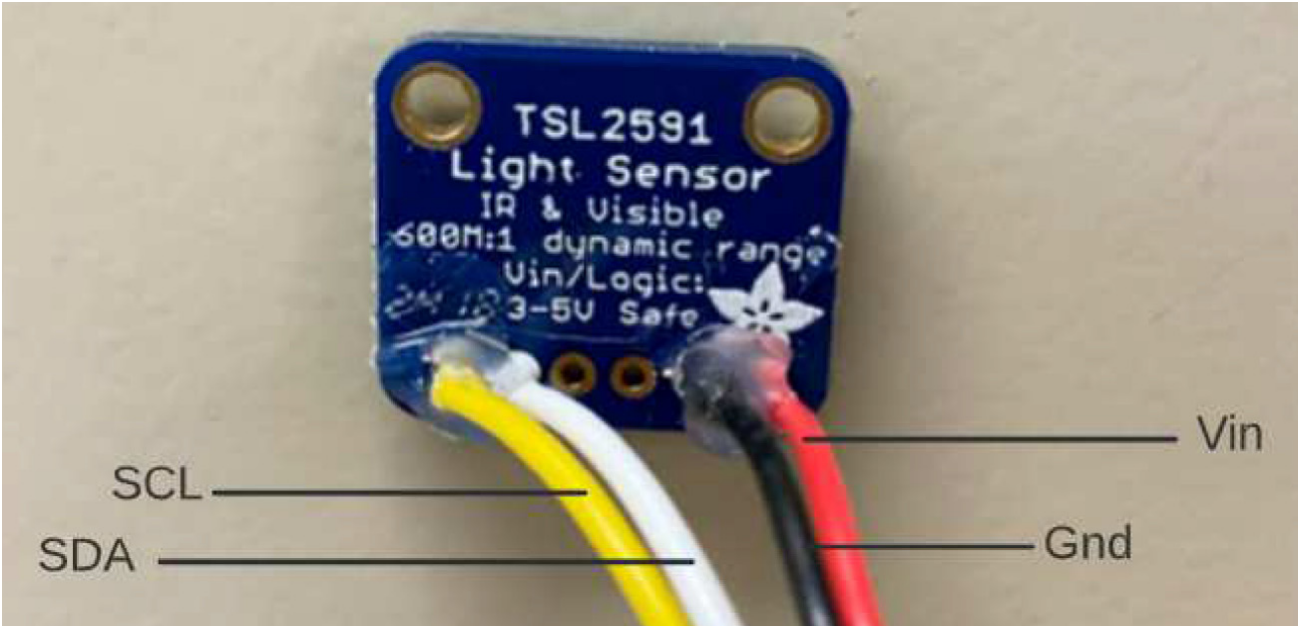
TSL2591 with JST wires connected.

**Fig. 15:**
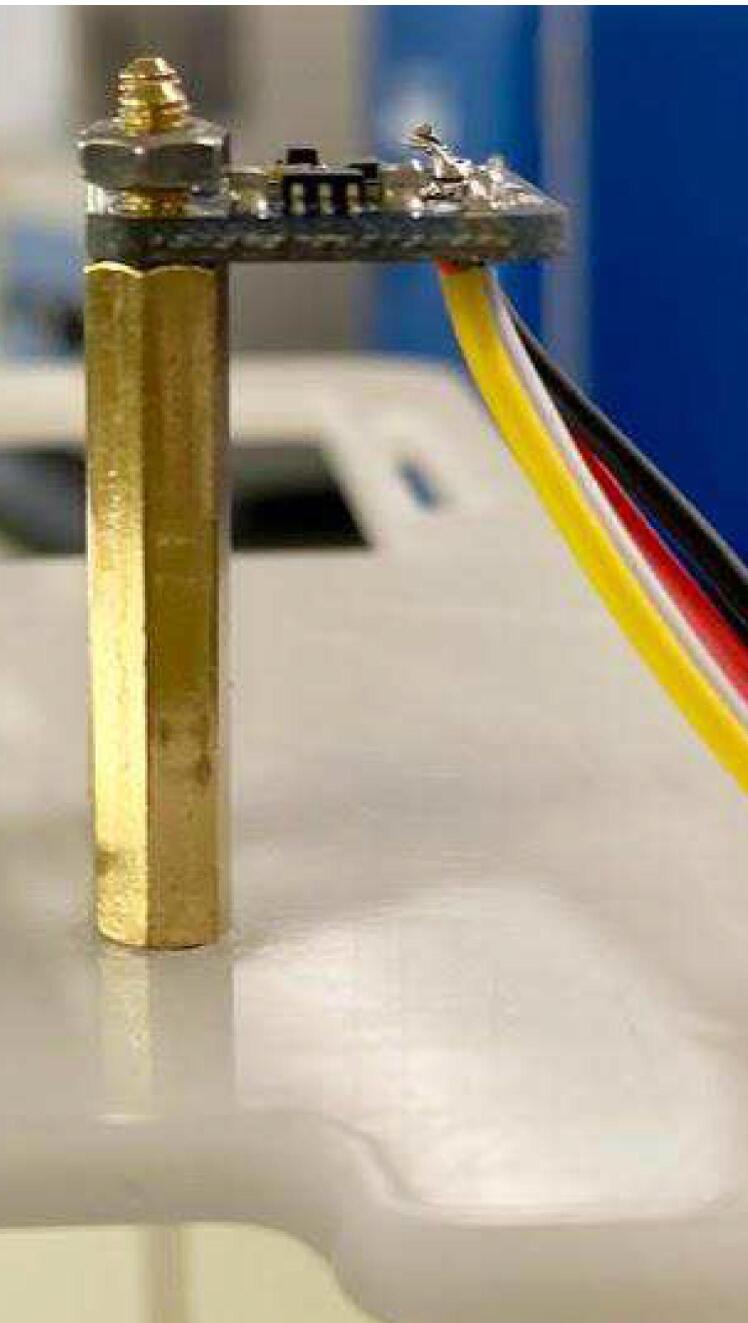
TSL2591 on hex standoffs.

Melt solder onto the Vin, Gnd, SDA and SCL holes. Following the wiring diagram, solder the 4-pin JST wires through the back of the TSL2591 (Fig. 14, Table 3). Apply hot glue over the wire connections. Attach the two hex standoffs projecting up to the case insert. Set the TSL2591 on top of the hex standoff with the sensor facing up, the two largest holes lined up with the standoffs, and the wires farthest away from the proto doubler. Use two M2.5 mm screws to secure the TSL2591 to the hex standoffs (Fig. 15).

**Table 3:** TSL2591 to JST wiring colors.

**Label**	**JST Wire Color**
SCL	Yellow
SDA	White
Vin	Red
Gnd	Black

### Case insert

Create the case insert by using a laser cutter and the STL file in the Zenodo repository. To install the case insert, make sure the screws for the proto doubler and hex standoffs have been screwed in before placing the case insert into the enclosure with the rounded edge on the same side as the cable glands. The battery will be fastened using the hook and loop strap through the laser cut holes on the case insert.

### PCB assembly

**Fig. 16:**
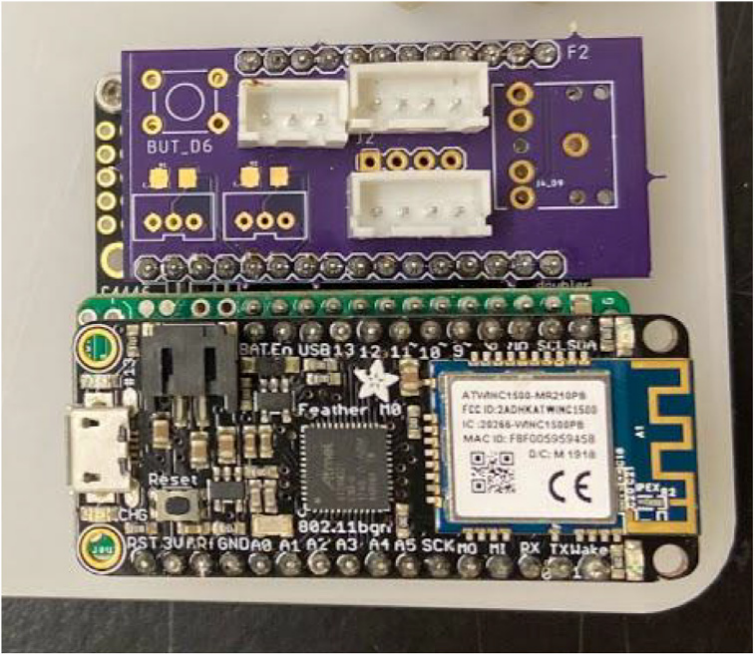
Assembled PCB stacks: Hypnos, Feather M0 WiFi, and Chime V1 PCB on the doubler.

Stack the Hypnos board on the left (closest to enclosure wall) side of the doubler using the headers (Fig. 15). Stack the Feather M0 WiFi on top of the Hypnos board using the headers (Fig. 16). Stack the Chime V1 PCB on the right side of the doubler using the headers (Fig. 16). Plug the female 3-pin JST connector from the GS3 into the male 3-pin JST connector on the PCB (Fig. 1). Plug the female 4-pin JST from the SHT30 into the male JST closest to the 12-pin header on the PCB (Fig. 1). Plug the female 4-pin JST from the TSL2591 into the male JST closest to the 16-pin header on the PCB (Fig. 1).

## Software setup instructions

### Mosquitto server script

In our use case, we found that utilizing a “local” instance of an MQTT broker (one in which we had full control) would be the best for this project. To accomplish this, we utilized an Open Source MQTT broker called Mosquitto. The basic directions for setting up Mosquitto can be found on the WeatherChimes Github repository README [26]. These instructions can be followed on a server or a desktop computer as long as the device in question can be accessed over the internet. This broker will connect to Node-RED which acts as a parser between MQTT packets and MongoDB.

### Setting up your MongoDB cluster

You will need to create a MongoDB account. The Node-RED server is a pass-through script to funnel the MQTT requests from Mosquitto into the MongoDB cluster for the project. A comprehensive guide to setting up your database is beyond the scope of this text, but can be found under the MongoDB resources online [27]. A replica set should be used for this cluster, instructions for setting this up are available in the MongoDB resources linked below. Once the cluster has been successfully deployed, you will need to retrieve some basic information. The main things we need to know are the cluster name, username and password to authenticate to the cluster. Next, we need to install Node-RED [28]. A detailed guide to connecting MQTT to MongoDB using Node-RED can be found on the WeatherChimes Github repository [26].

For the WeatherChime hardware Arduino programming, we will need to specify the hostname on which the Mosquitto broker is listening as well as the username and password that the broker is using. In addition, you will need to provide a “Site Name”, this corresponds to the database in the MongoDB cluster where the data will be stored, all of this is specified in the “arduino_secrets.h” located in the WeatherChimes Git repository.

### Software installation

Before code can be uploaded to the Feather, download the Arduino IDE software [29] and set up the board profile to include the necessary libraries such as Loom [30]. TSL2591, SHT30 and SDI-12 implementations are all provided within the standard Loom library. Instructions for completing these steps are present in the OPEnS Loom Quick Start Guide [31]. The steps for completing the specifics of the WeatherChimes installation are on Github [26] under the “Loom Installation and MQTT Integration” section.

### Sensor placement and installation

To ensure proper operation of this device, locate an area where the device enclosure will not receive too much sunlight for the light sensor and stable WiFi internet connection. The TSL2591 datasheet states sensitivity from 0.000118 to 88,000 Lux [22]. On a normal, clear day the average Lux levels are approximately 10,000 Lux to 25,000 Lux (indirect sunlight). While in direct sunlight, Lux levels can fluctuate between 32,000 to 130,000 Lux. Depending on the weather condition, it is recommended to look up average Lux levels for that weather and compare with the Lux levels being logged and adjust location accordingly if need be. We were unable to derive a direct translation of the Lux value into a more common W/m^2^ unit, but an approximate conversion for sunlight of 0.0079 W/m^2^ per Lux has been suggested in other applications [32]. Informal testing of the TSL2591 sensor with a commercial pyranometer showed lower than expected levels in W/m^2^ after conversion was applied, so discrete calibration of each TSL2591 to a commercial pyranometer is advised if W/m^2^ is desired. An average RSSI value for good internet connection is −50 dBm to −75 dBm [33]; anything lower and most online activities will be impacted.

The Pelican case is watertight and dustproof, as well as resistant to cracking. When looking for an area to place the device, avoid areas that lack water drainage, as well as places that might have possible falling objects. The maximum temperature that the device is able to handle before overheating is 199°F [34], so putting it in direct sunlight should not cause any issues. However, this may lead to the TSL2591 sensor to be oversaturated.

**Fig. 17:**
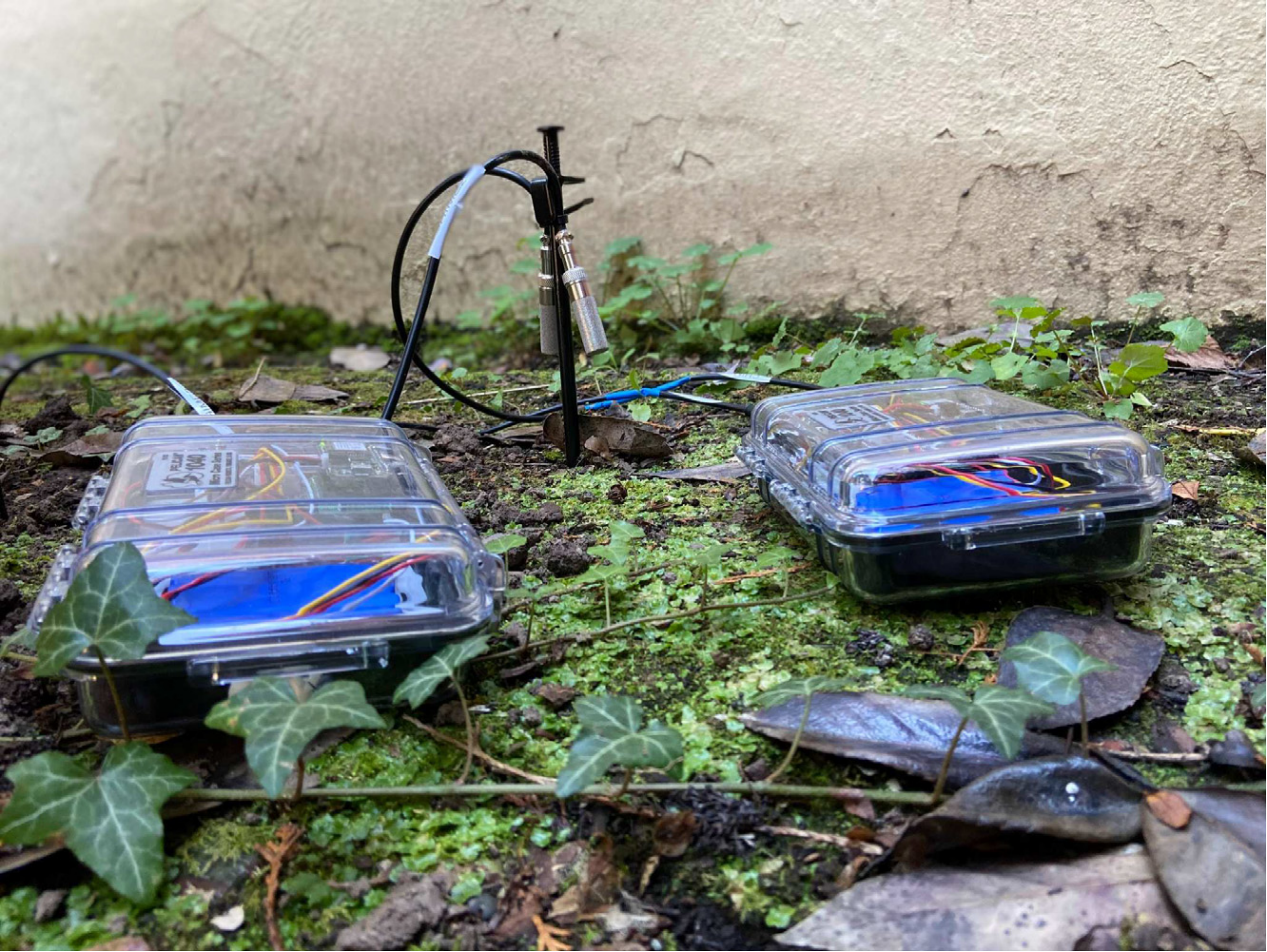
SHT-30 sensors zip-tied to tent stake.

The SHT30 sensor should not be in contact with the ground and ideally vertical to measure and log data properly. For near ground installation, a hose clamp or zip tie could be used to attach the sensor to a tent stake with the sensor below the cable (Fig. 17).

For the METER GS3 soil sensor, a hole roughly 6 in. wide should be dug to several cm below the depth of interest. The probes should be inserted horizontally into the side of the hole at the depth of interest with the long side of the white sensor body vertical and the cable at the top. Fill back in the hole. Care should be taken during installation to optimize contact between the sensor probes and soil to prevent air pockets and soil compaction between probes. The METER GS3 manual [24] provides additional installation considerations including avoiding installation adjacent to large metal objects, and spacing for multiple sensors. For best results, the soil sensor should be inserted in damp soil to get accurate readings for electrical conductivity. When removing the sensor, do not lift it from the ground by the cable as this could damage the internal connections of the device. Instead, use a tool to dig around the sensor and pull it up by the white sensor body.

### Sensor calibration

Individual sensor testing should be conducted to verify and validate the functionality of all sensors against a known reference before they are integrated into a complete device. This may be conducted with industry-standard probes or other recently-calibrated sensors. During this phase, each sensor that will be present on the entire system is individually plugged into the main PCB, to ensure other sensors do not interfere. After each sensor is tested, all sensors are tested together on the system to ensure functionality.

### Launching the device

Set the battery in the case insert with the wire in the corner closest to the TSL2591, and secure it with the hook and loop strap. Add desiccant to the Pelican case on top of the battery. Plug the battery into the black JST connector on the Feather M0. Verify that the WiFi light on the Feather and LED on the Hypnos flash; they will periodically flash with each data transmission. Close the enclosure ensuring that nothing is interfering with the gasket. Log in to the MongoDB to verify data with appropriate values are being logged for each sensor and the battery.

### Max8 application and accessing MongoDB data

Max8, or MaxMSP (Max Signal Processing), by Cycling74 is an object-oriented, modular, graphical programming environment [35]. Resembling MIT’s Scratch [36] and LabView by National Instruments [37], Max’s approach to coding includes drag-and-drop functionality and connecting components via patch cords. A custom package called Loom4Max was designed by the authors for extending the Max8 software application for use with live sensors.

Source files for the Loom4Max package as of the date of publication are also on this paper’s associated Zenodo repository, though it is recommended to use the latest version on GitHub. Instructions for installing Max and the Loom4Max package can be found at the Loom4Max wiki [38].

**Fig. 18:**
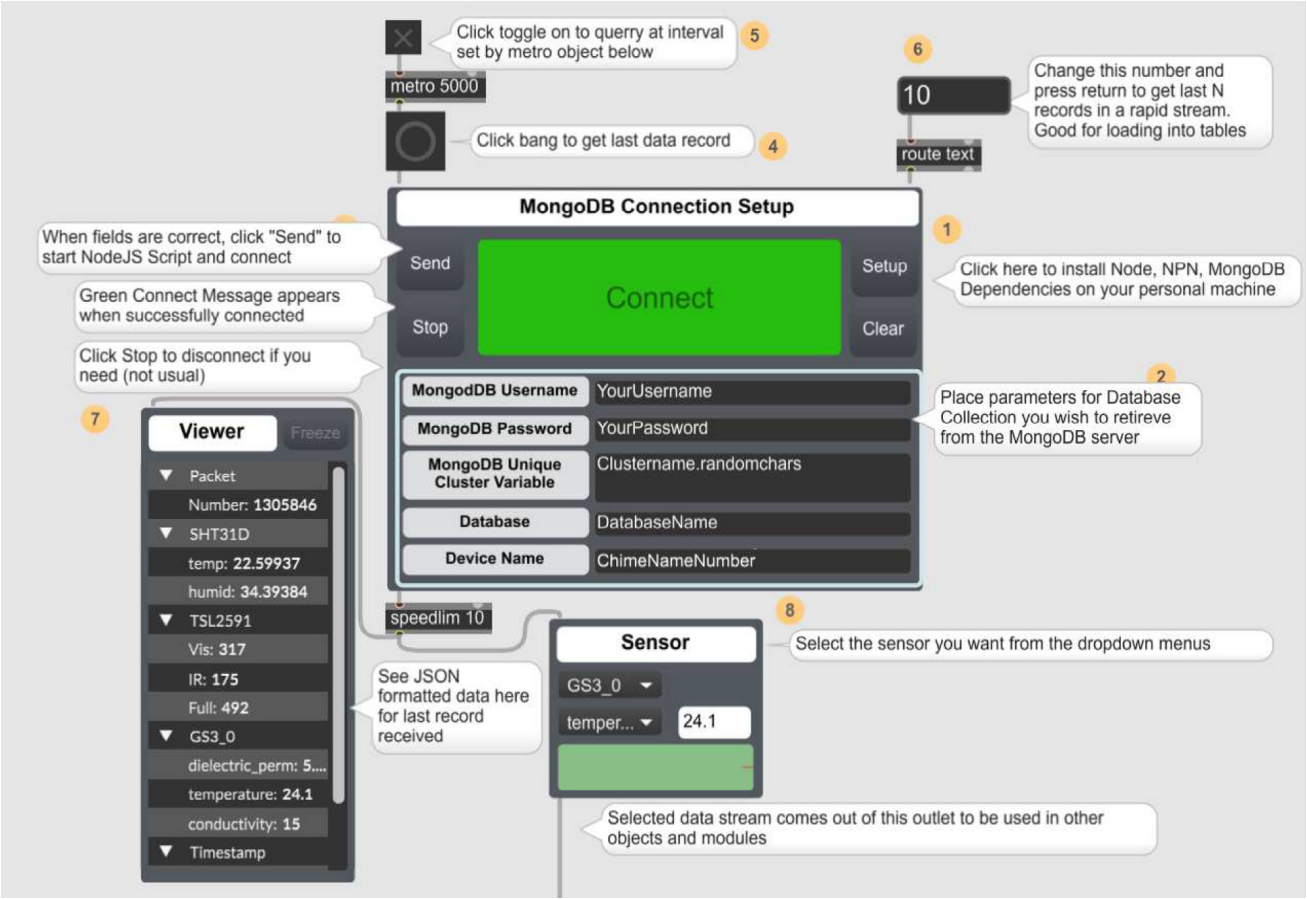
Example connecting to MongoDB using Loom4Max Package.

After installation, you can access a demo for connecting to a WeatherChimes device by navigating in the Max menu to Extras > Loom > 00.Sensors > 02.MongoDBConnect, which will bring up the example shown in (Fig. 18).

A user can connect to a MongoDB database for real-time access by following the sequentially numbered comments in the WeatherChimes example (Fig. 18). The box labeled MongoDB Connection Setup is the module that contains the NodeJS scripts and helper functions that input credentials to log into a specific database. This is called “subscribing” to the database. On connection, the most recent data packet published by the WeatherChime will be outputted into the Max environment as JSON formatted data for use. While connected, newly-published packets will be immediately updated in Max. The user can request the most recent packet at any time (Fig. 17**, comment 4**). They can also request the last N packets of the database (Fig. 18**, comment 6**) to be outputted for plotting, manipulation, and playback. Helper modules in the Weather Chimes example include *Viewer* (Fig. 18**, comment 7**), which displays the JSON data in a readable form; *Sensor* (Fig. 18**, comment 8**), which enables selecting and parsing of a specific sensor in the data packet for usage; and *Rescale* (Fig. 18**, comment 9**), which linearly remaps an incoming minimum–maximum data range into a new min–max range to control musical or visual effect parameters that will likely expect a different range of numbers (e.g. sound volume parameter may expect integers between 0 and 127).

## Validation and characterization

**Fig. 19:**
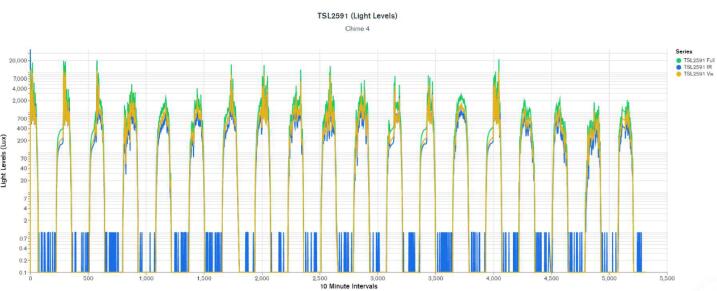
MongoDB chart displaying TSL2591 Lux data.

**Fig. 20:**
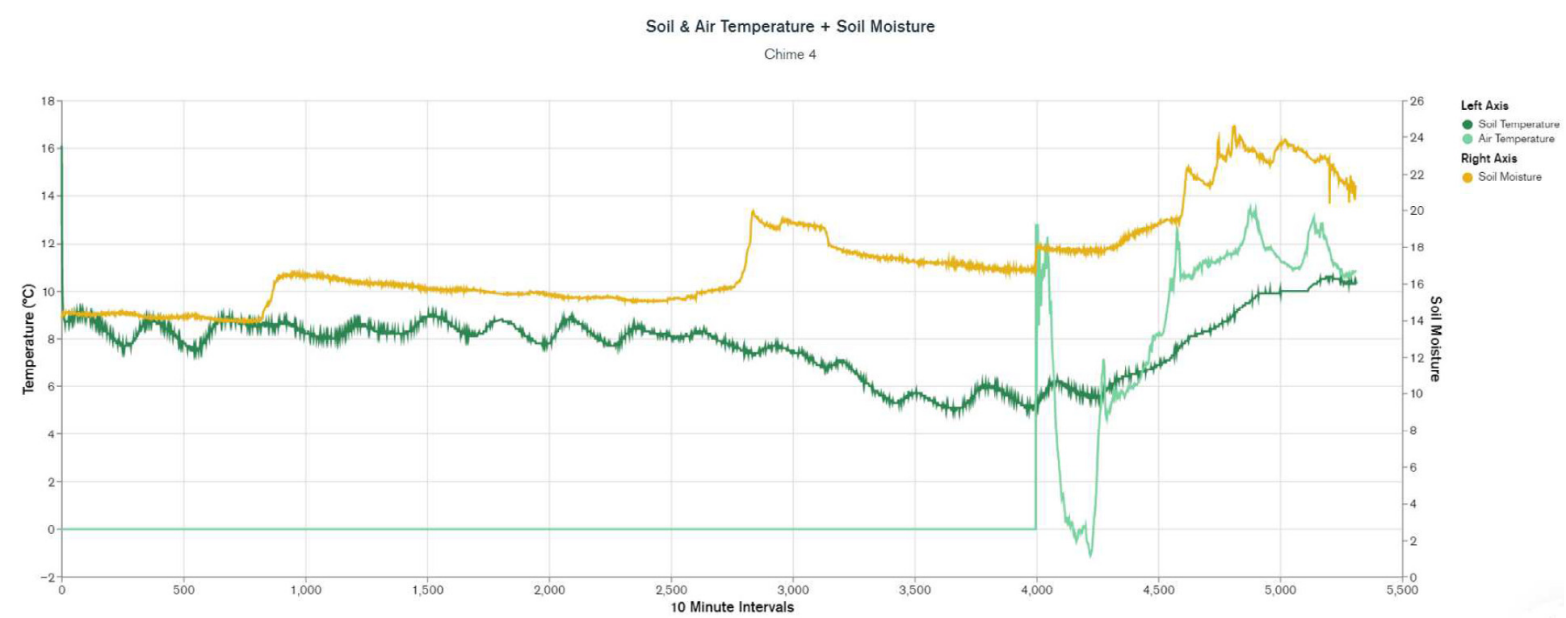
MongoDB chart displaying GS3 VWC, Temp and SHT30 Temp.

**Fig. 21:**
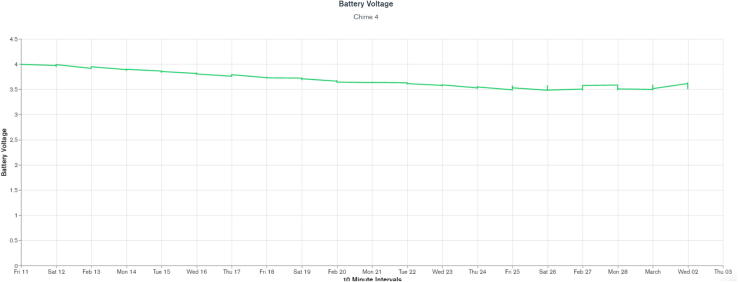
MongoDB chart displaying battery level.

**Fig. 22:**
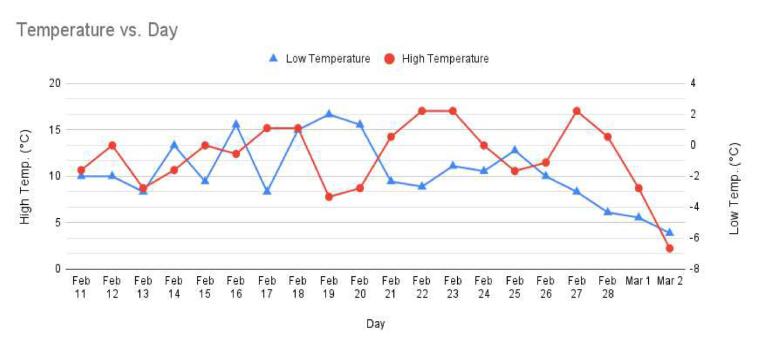
Low and high temperature in Corvallis from Feb. 11 to Mar. 2.

**Fig. 23:**
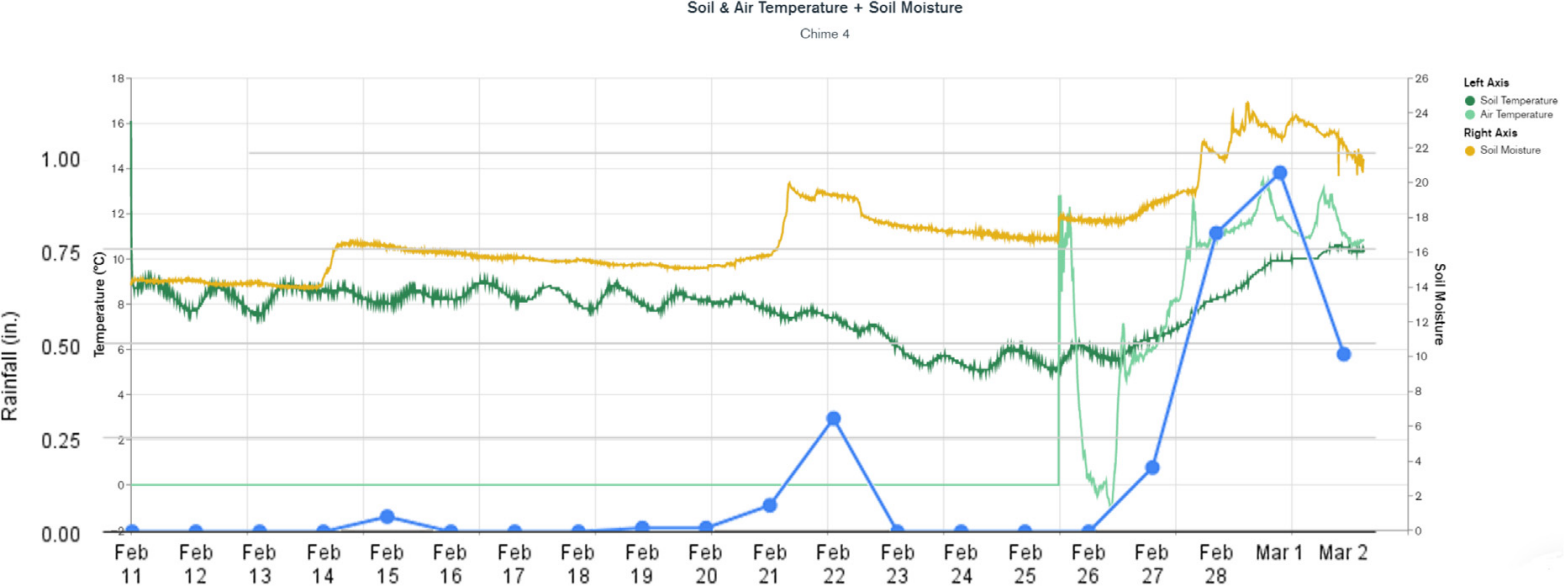
Rainfall overlapped with soil moisture MongoDB chart.

WeatherChimes was tested as part of an Honors College class at Oregon State University, Corvallis, OR. For convenience, it was deployed by the students on campus at a site that possessed both environmentally dynamic and artistically interesting qualities, and had access to the campus WiFi signal. The southwest corner of Strand Hall was chosen, a building facing the campus quad with ample south-facing and afternoon light, featuring a statue of a pioneer woman and a bike rack. Students installed the WeatherChimes sensor at the site. A garden shovel was used to dig a hole that was at least 6 in. deep, with the soil moisture sensor installed horizontally into the dirt. Next, a tent stake was driven into the dirt near the case. The JST connector for the battery was plugged into the Feather M0 and checking MongoDB confirmed successful data transmission. We plotted data for 19 diurnal cycles from February 11, 2022 to March 2, 2022 to plot full, visible and infrared light (Fig. 19). Infrared light was detected in nighttime periods due to lighting fixtures at the site. The SHT30 sensor was installed 13 days later, which is why there are no readings for air temperature and humidity until day 13. Fig. 20 shows soil VWC and temperature, and a portion of air temperature over the sampling period. Fig. 21 shows lithium polymer battery depletion from close to fully charged at 4.2VDC down to 3.5VDC where it terminates operation. These voltages are then shifted to the appropriate voltage to power the Feather M0. While we shut sensors down and put the microprocessor to sleep in between sample cycles to conserve power, we discovered after the deployment the setting for the WiFi radio was not in low-power mode. We believe the system would last significantly longer with better power management of the radio, or solar charging added. In determining sensor accuracy of the system, we compared our sensor plots to recorded weather data of Corvallis, Oregon between February 11, 2022 to March 2, 2022 in which the system was deployed [39], [40]. Fig. 22 and Fig. 23 reflect the temperature, humidity and precipitation plots from local weather data compared to our system.

Based on this data, we conclude our sensors agree with locally-recorded meteorological events. Fluctuations in soil moisture correlate with reported rainfall. The slight disjunction between soil moisture and rainfall on the plot can be explained due to rainfall being reported on a daily schedule, opposed to the ten minute schedule for soil moisture. Air temperature of our system co-varies alongside reported highs and lows for the area. Highs, and especially lows, do not match exactly due to our installation location near the south side of a brick building, which may have prevented the temperature sensor from reporting as low as the meteorological data.

### Sonification and visualization in Max8

The Honors College class collected sound, video, and pictures of the site using media recorders. They were tasked with using this media as subject material for data visualization and sonification. The environmental data was used to shape the sounds and visuals of the site from which it came.

### Pioneer woman

**Fig. 24:**
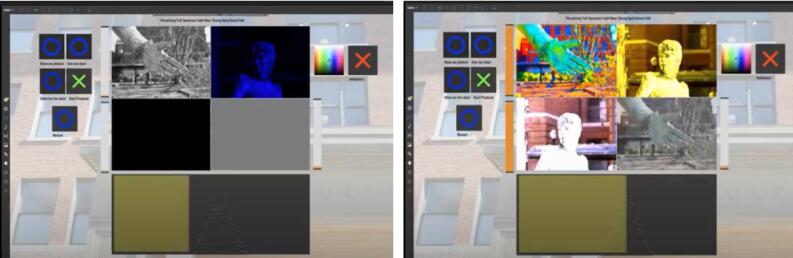
Electric Nature Interface.

*And Then There Was Light (Sometimes)* (Fig. 24) is a visual installation by student Brandt Bridges [41]. The student photographed the pioneer woman statue from different angles. Sunlight data from the past 24hrs was read into a table and played back (values output sequentially at a defined rate) in a looping pattern. Data values were rescaled to control visual effects in four different views of the pioneer woman. This subject was selected because the pioneer woman is symbolic of what it is like to stand out at the site. The relationship between the data and visualization were intended to create a connection between the solar radiation falling on the pioneer woman and the color bias, saturation, brightness, and contrast of the images.

### Sonification

**Fig. 25:**
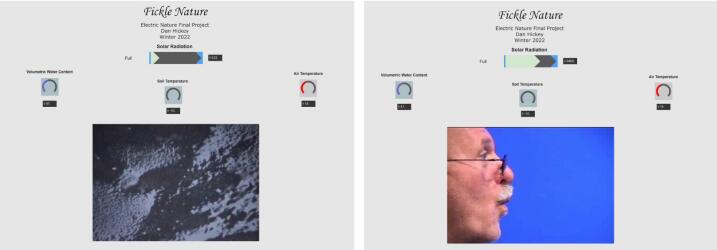
Fickle Nature interface.

Daniel Hickey used sound samples from the site to create an algorithmic music generator called *Fickle Nature* (Fig. 25) [42]. He used sunlight, soil moisture, soil temperature, and air temperature as parameters to control which musical layers will be present in the mix. There are two opposing musical tracks, each different in style: dark/ominous versus upbeat/happy. Which one is played depends on the amount of visible light in the environment. If light is below a certain threshold, dark/ominous tracks will be activated, and vice versa. There is an animation in the center that changes based on the same light threshold and also rotates faster/slower based on the soil temperature. Within each track, certain instruments/patterns are added/removed based on data from the other sensors. For the ominous track, higher volumetric water content results in a “busier” beat, whereas the air temperature influences that same aspect of the happy track. Instruments used in each track were processed recordings from sounds collected at the field site.

### Sitka Sound Science Center

**Fig. 26:**
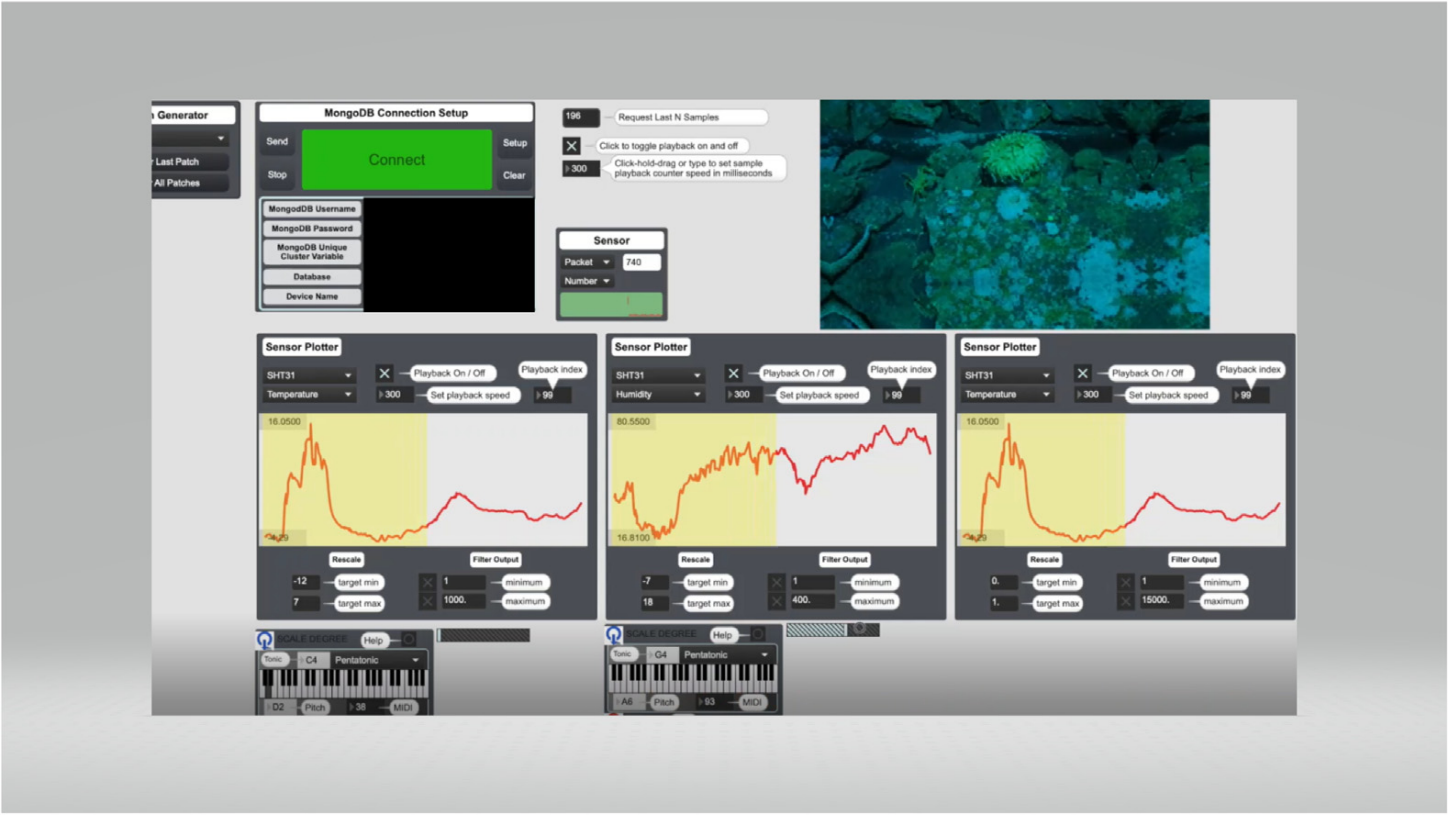
WeatherChimes interface showing data plots from the Sitka Sound Science Center.

Chet Udell, a professor at Oregon State University, demonstrated WeatherChimes at the Whalefest 2022 Workshops hosted at the Sitka Sound Science Center in Sitka, Alaska on November 2nd – November 7th 2022. WeatherChimes hardware was installed on the south side of the Sitka Sound Science Center building days in advance of the workshop. Video [43] of exhibits inside of the science center were collected, including the touch tanks containing star fish, anemones, and urchins was also recorded in advance. Udell conducted 3 workshops with undergraduate students, high schoolers, and community members using thirteen WeatherChimes kits indoors as an introduction to sensor technologies. Data sonification and visualization activities using the realtime data from the WeatherChime installed on the south side of the building followed. Fig. 26 below shows the MongoDB connection setup on the top-left, which establishes a connection with a specific data cluster based on credentials provided by the user. Samples over a given period may be requested from this interface. Data is played back and looped at a rate defined by the sample playback counter. The Sensor Plotter interfaces below show plots for the SHT30 air temperature (left and right graphs) and relative humidity (middle graph) data over the requested 48-hour period. Datasets from each sensor in the WeatherChimes system may be selected from the dropdown menus on these Sensor Plotter boxes. Minimum and maximum values of the Y axis for each plot are displayed on each graph. The keyboard boxes on the bottom-left translate the associated incoming data stream into melodies based on the Key and Scale settings on their interface. In this example, higher-value data will play a higher pitch on the scale, and lower-value data will play lower pitches. The red balance of the touch tank video (top-right) is determined by the data in the plot on the right; higher-value data increases red balance, and vice versa for lower-value data. This is one of many possible configurations for relating environmental sensor data to music and visual elements. Video and audio clips demonstrating this interface are included in the Zenodo repository.

Workshop participants reported the sonification and visualization exercises provided an insightful and engaging introduction to environmental sensors and experiencing their interrelationships using multiple senses. One user shared that hearing something like temperature expressed as a melody turned observing data on a plot into a time-based experience, becoming more aware of nuances of movement as the temperature unfolded. Another participant shared that being able to choose the scale of the melody to determine if it sounded happy or sad prompted them to think carefully about the meaning we ascribe to data and why we use data to communicate with others. Addition of other sensors like rain collectors, tipping buckets, and air quality sensors were recommended.

## Conclusion

In-situ environmental sensing systems have been created largely to automate measurement tasks that would have otherwise needed to be performed by hand on-site. This capability poses wide-ranging advantages and utility for advancing data-driven science and exploring timescales and precision beyond hand-made measurements. The capability to upload and access data directly over servers online has also decreased the need to interact with field sites to retrieve data and monitor system performance. WeatherChimes demonstrates this base functionality in an open-source package released for the broader use of the scientific community. It also extends these applications to represent and explore data through other sensations and modalities including music and visual art. Integrating sonification and alternative visualization tools into an IoT data logging platform may not only yield insights into data that could not be as easily perceived from a graph at a glance, but may help users relate with and ascribe meaning to the data in new ways. Other advantages of this system include cost savings and portability.

In the near future, we hope to provide 4G internet connectivity, decrease power consumption for longer deployment periods, and explore integrating other sensors including barometric pressure, air quality, and rainfall. We also aim to collect user experience and software administration surveys to yield data that will inform our design decisions into future embodiments of the system and ensure the needs of our target community are being addressed.

## Ethics statement

The work does not use any human or animal subjects.

## Declaration of Competing Interest

The authors declare that they have no known competing financial interests or personal relationships that could have appeared to influence the work reported in this paper.

## Figures and Tables

**Table:** 

Operation Mode	Measured Current Draw (mA)	Time period (Seconds)
Initialization	107.5	1.4
WiFi/Sensor Measurements/SD Logging	108.9	1.8
Sleep/Standby	14.8	600

## Appendix A Power consumption calculations

*Battery self-discharge:*

Battery Life Calculated using 10,500 mAh battery capacity each.

BatterySelfDischargeRate = $\frac{\mathrm{Self}\;Disch\arg e\;Rate\;Per\;Month}{730}$

BatterySelfDischargeRate = $\frac{0.05*10500}{730}=0.719\;mA$

CurrentDraw = $\frac{\Sigma Current\;Draw*Time\;Period}{\Sigma Time\;Period}+Self\;Disch\arg e\;Rate$

CurrentDraw = $\frac{\left(107.5*1.4\right)+\left(108.9*1.8\right)+\left(14.8*600\right)}{1.4+1.8+600}+0.719\;mA=16.015\;mA$

BatteryLife = $\frac{\mathrm{Battery}\;Capacity}{\mathrm{Current}\;Draw}$

BatteryLife = $\frac{10500}{16.015}=655.637\;hours=27.32\;days$

Table A1. Current draw of node during operating phases.
